# Simultaneous Resection for Type A Thymoma With Synchronous Solitary Pulmonary Metastasis: A Case Report

**DOI:** 10.1002/rcr2.70512

**Published:** 2026-02-18

**Authors:** Takamitsu Hayakawa, Mikako Mitake, Hirohisa Inaba, Saita Miyamoto, Takuya Masuda, Shingo Takahashi, Atsuki Fukada, Ai Sugimoto, Masako Morita, Hiroyuki Matsuda, Asako Okabe, Kazuhito Funai

**Affiliations:** ^1^ Department of Thoracic Surgery Japanese Red Cross Shizuoka Hospital Shizuoka Japan; ^2^ Department of Respiratory Medicine Japanese Red Cross Shizuoka Hospital Shizuoka Japan; ^3^ Department of Diagnostic Pathology Japanese Red Cross Shizuoka Hospital Shizuoka Japan; ^4^ First Department of Surgery Hamamatsu University School of Medicine Hamamatsu Japan

**Keywords:** complete resection, metastatic pulmonary tumour, type a thymoma

## Abstract

Type A thymoma is associated with an excellent prognosis, and distant metastasis is uncommon. Optimal management for metastatic type A thymoma has not been well documented. We report a case of conventional type A thymoma with synchronous solitary pulmonary metastasis treated with simultaneous resection. An 81‐year‐old woman was incidentally found to have both a 2.0 cm anterior mediastinal nodule and a 0.9 cm pulmonary nodule in the left lower lobe. Both lesions were resected via video‐assisted thoracoscopic surgery. Histopathology and immunohistochemistry revealed identical features in both tumours, confirming type A thymoma with solitary pulmonary metastasis (pT1aN0M1b, stage IVb). No histopathological features of atypical type A thymoma were present. The patient remains recurrence‐free 1 year after surgery without adjuvant therapy. This case suggests that pulmonary metastasis can occur even in small, low‐grade type A thymoma and that simultaneous resection for diagnostic and therapeutic purposes may be considered to achieve favourable outcomes.

## Graphical abstract

We report a case of conventional type A thymoma with synchronous solitary pulmonary metastasis treated with simultaneous resection. This case suggests that pulmonary metastasis can occur even in small and low‐grade type A thymoma. Simultaneous resection for diagnostic and therapeutic purposes may be considered to achieve favourable outcomes.

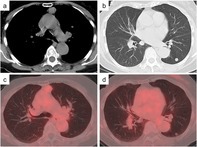

## 1 Introduction

Type A thymoma is considered the thymoma subtype with the most favourable prognosis and the lowest malignant potential, and distant metastasis is rare [1]. Therefore, few reports have described treatment for Type A thymoma with distant metastasis. Herein, we report a case of simultaneous resection of the primary lesion and a solitary synchronous pulmonary metastasis from a Type A thymoma.

## 2 Case Report

**FIGURE 1:**
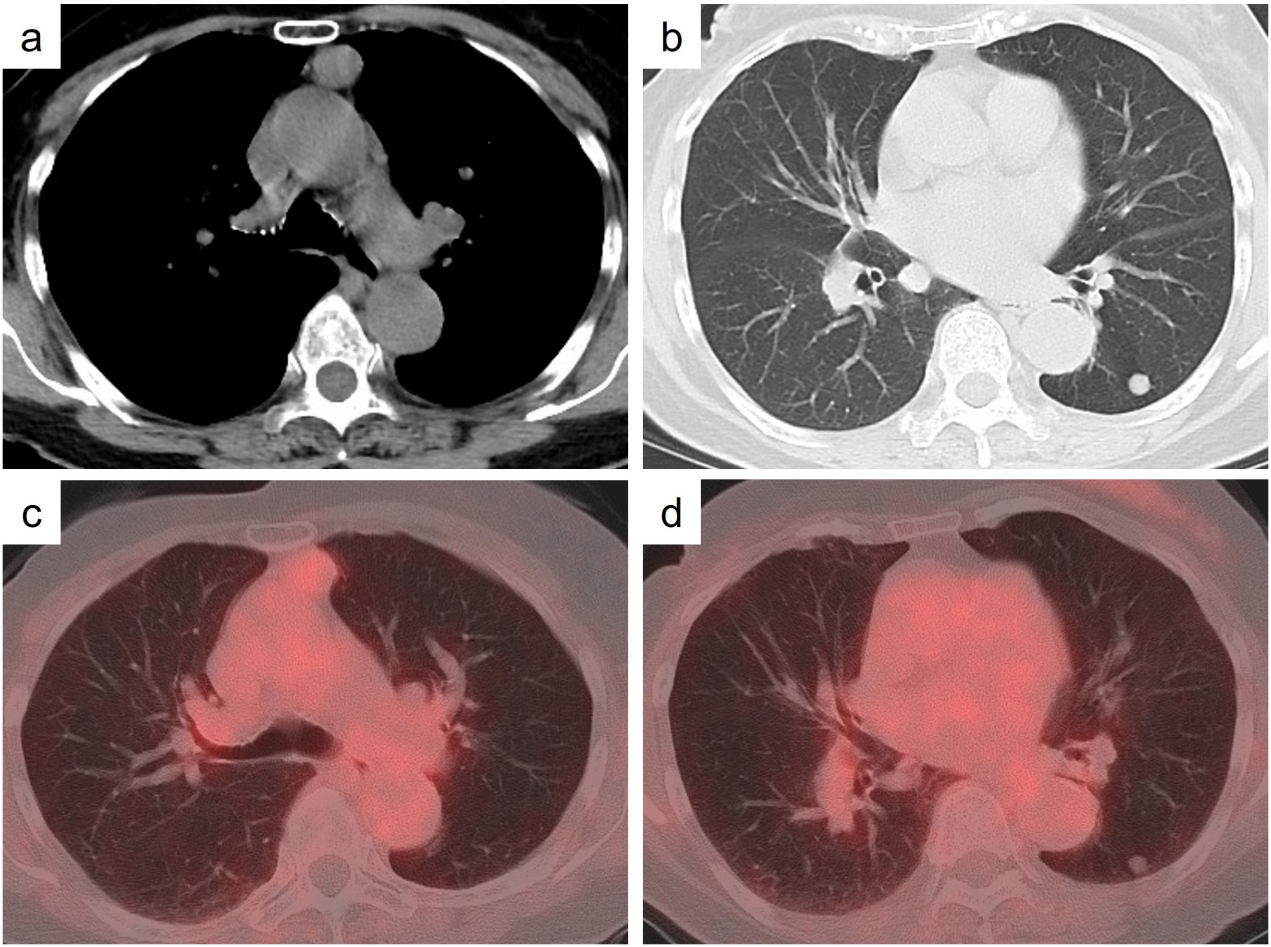
Radiological findings. Chest computed tomography shows (a) a well‐defined nodule in the anterior mediastinum and (b) a solid nodule in the left lower lobe. Positron emission tomography reveals (c) slight accumulation on the anterior mediastinal nodule and (d) no accumulation on the nodule in the left lower lung.

The patient was an 81‐year‐old woman with no smoking history. She had a history of right breast cancer treated approximately 20 years earlier. Chest computed tomography (CT) incidentally revealed a well‐defined 2.0 cm nodule in the anterior mediastinum and a well‐defined 0.9 cm solid nodule in the left lower lobe (Figure 1a,b). Positron emission tomography (PET) demonstrated uptake in the anterior mediastinal nodule with a maximum standardised uptake value of 2.13, whereas no uptake was observed in the left lower lobe nodule (Figure 1c,d). The serum anti‐acetylcholine receptor antibody level was not elevated. The anterior mediastinal lesion was preoperatively diagnosed as thymoma, while the left lower lobe nodule was suspected to be a benign tumour or pulmonary metastasis from prior breast cancer. Resection of both nodules was planned for diagnostic and therapeutic purposes.

First, for the left lower lobe nodule, we performed thoracoscopic‐assisted partial resection of the left lower lobe using a port at the seventh intercostal space on the anterior axillary line and a 6 cm mini‐thoracotomy directly above the tumour. Subsequently, an additional port was placed at the third intercostal space on the anterior axillary line, and thoracoscopic thymectomy was performed. The postoperative course was uneventful, and the patient was discharged on postoperative day 4.

**FIGURE 2:**
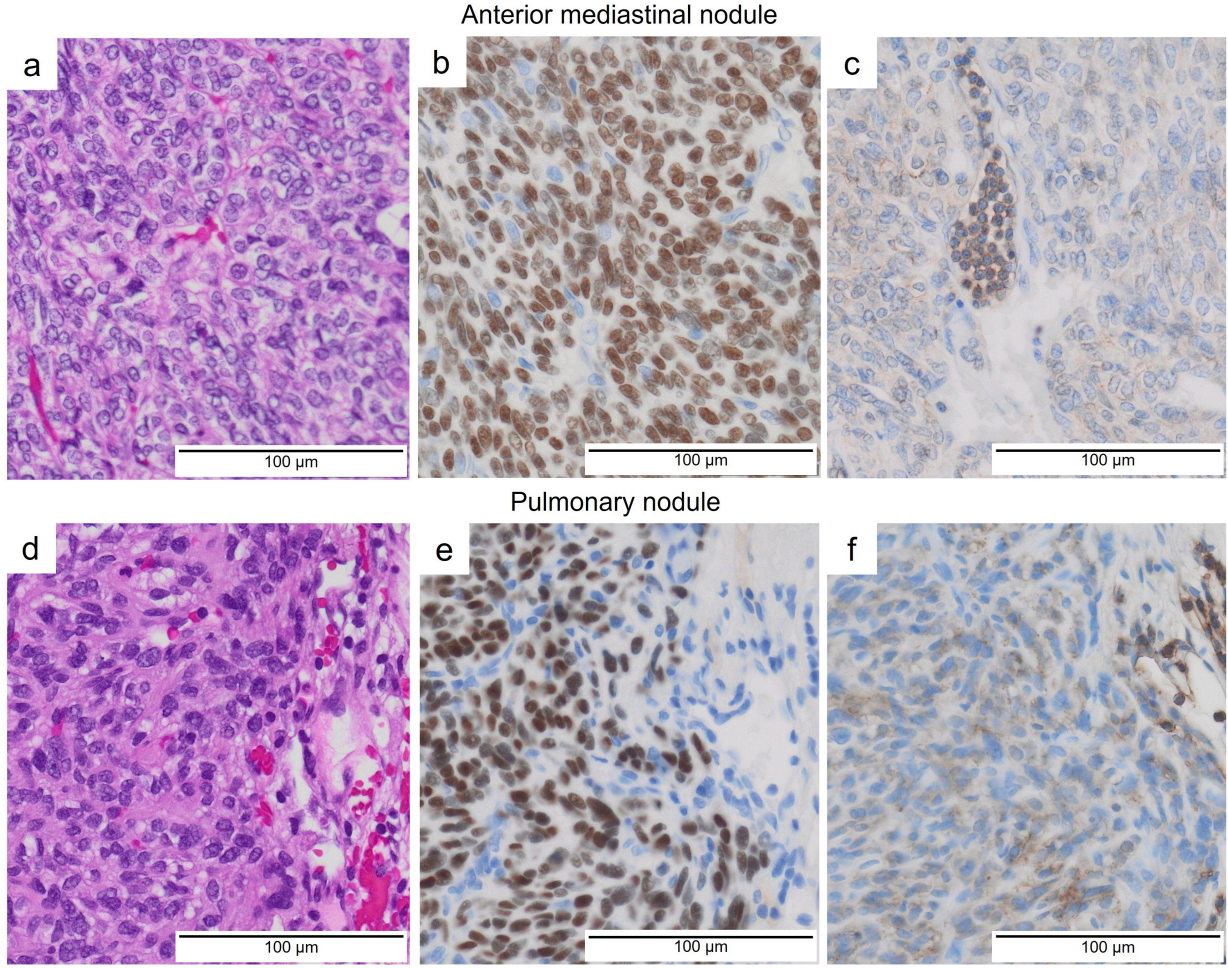
Histopathological findings. In the anterior mediastinal nodule, (a) the haematoxylin–eosin stain demonstrates spindle cell proliferation with a fascicular pattern. (b) p40 is positive. (c) CD99‐positive lymphocytes are focally present. In the pulmonary nodule of the left lower lobe, (d) the haematoxylin–eosin stain demonstrates spindle cell proliferation. (e) p40 is positive. (f) CD99‐positive lymphocytes are scarcely identified.

Macroscopically, both tumours were solid and whitish. Histopathologically, the anterior mediastinal nodule consisted of nests of spindle cells with small oval nuclei, with few lymphocytes (Figure 2a). Capsular invasion was present, but invasion into surrounding thymic tissues was not observed. Immunohistochemically, the tumour cells were positive for cytokeratin 5/6 and p40 (Figure 2b). CD99‐positive lymphocytes were focally present (Figure 2c). The left lower lobe nodule showed morphological and immunohistochemical findings largely similar to those of the anterior mediastinal tumour (Figure 2d–f). The pulmonary nodule was negative for GATA‐binding protein 3, ruling out recurrent breast cancer. Based on these findings, the final diagnosis was Type A thymoma (pT1aN0M1b, stage IVb, TNM classification 9th edition). Because complete resection was achieved, no adjuvant therapy was administered. The patient remains recurrence‐free 1 year after surgery.

## 3 Discussion

In this case, a type A thymoma—generally considered to have a favourable prognosis—demonstrated pulmonary metastasis. Resection of both the primary and metastatic lesions resulted in disease control without recurrence. These findings suggest that even in stage IVB type A thymoma with distant metastasis, surgical resection may be effective.

Predicting poor prognosis in type A thymoma is difficult. In general, type A thymoma is the thymoma subtype with the lowest malignant potential, and distant metastasis is rare [1]. In contrast, an atypical variant of type A thymoma has been described. This variant exhibits monoclonal proliferation of spindle‐shaped or oval cells similar to conventional type A thymoma, but with histological features such as hypercellularity, increased mitotic counts, and focal necrosis [2]. Several reports have described distant metastases, primarily to the lung, and have suggested that this variant may confer a poorer prognosis [3, 4]. Because these histological features were absent, the present case was classified as a conventional type A thymoma. The primary tumour was small, lacked invasion of surrounding tissues, and demonstrated low PET uptake; thus, no clinicopathological factors predictive of distant metastasis were identified. Even when imaging suggests a low‐grade thymoma, the possibility of pulmonary metastasis cannot be excluded, and resection of concurrent pulmonary lesions should be considered for both diagnosis and treatment.

For resectable stage IVb thymoma, achieving total resection, including metastatic lesions, is important for a favourable prognosis. In advanced thymoma, cases with macroscopic complete resection have significantly better outcomes than those without [1]. Furthermore, among cases with complete resection, postoperative adjuvant therapy did not improve outcomes, and prognosis was equally favourable regardless of whether adjuvant therapy was administered [1]. Because total resection was achieved, a relatively favourable prognosis can be expected even without adjuvant therapy in this case. Recurrence patterns of thymic epithelial tumours include pleural, pulmonary, local, bone, and hepatic recurrence [5]. Solitary pulmonary metastasis is a type of distant metastasis that is relatively achievable to complete resection, and surgical resection should be considered. In thymoma, repeated resection for recurrent lesions has been suggested to improve survival [5]. Thus, if asynchronous pulmonary metastasis develops in this case, re‐resection would be a potential treatment option.

In conclusion, because type A thymoma can present with pulmonary metastasis, concurrent pulmonary nodules should be resected for both diagnosis and treatment. In the present case, complete resection was achieved, and a relatively favourable prognosis can be expected.

## Author Contributions

T.H. drafted the manuscript. M.M.1, H.I., S.M., T.M., S.T., A.F., A.S., M.M.2, H.M., A.O., and K.F. revised the manuscript accordingly. T.H., M.M.1, H.I., and S.T. managed patients. All the authors approved the final version of the manuscript.

## Funding

The authors have nothing to report.

## Consent

The authors declare that written informed consent was obtained for the publication of this manuscript and accompanying images using the consent form provided by the Journal.

## Conflicts of Interest

The authors declare no conflicts of interest.

## Data Availability

Data sharing not applicable to this article as no datasets were generated or analysed during the current study.
